# Serum Cystatin C Reflects Angiographic Coronary Collateralization in Stable Coronary Artery Disease Patients with Chronic Total Occlusion

**DOI:** 10.1371/journal.pone.0137253

**Published:** 2015-09-24

**Authors:** Ying Shen, Feng Hua Ding, Rui Yan Zhang, Qi Zhang, Lin Lu, Wei Feng Shen

**Affiliations:** 1 Department of Cardiology, Rui Jin Hospital, Shanghai Jiaotong University School of Medicine, Shanghai 200025, People’s Republic of China; 2 Institute of Cardiovascular Diseases, Shanghai Jiaotong University School of Medicine, Shanghai 200025, People’s Republic of China; Universitätsklinikum des Saarlandes, GERMANY

## Abstract

**Objective:**

We investigated whether and to what extent cystatin C was associated with angiographic coronary collateralization in patients with stable coronary artery disease and chronic total occlusion.

**Methods:**

Serum levels of cystatin C and high-sensitive C-reactive protein (hsCRP) and glomerular filtration rate (GFR) were determined in 866 patients with stable angina and angiographic total occlusion of at least one major coronary artery. The degree of collaterals supplying the distal aspect of a total occlusion from the contra-lateral vessel was graded as poor (Rentrop score of 0 or 1) or good coronary collateralization (Rentrop score of 2 or 3).

**Results:**

In total, serum cystatin C was higher in patients with poor collateralization than in those with good collateralization (1.08 ± 0.32 mg/L *vs*. 0.90 ± 0.34 mg/L, P < 0.001), and correlated inversely with Rentrop score (adjusted Spearmen’s *r* = -0.145, P < 0.001). The prevalence of poor coronary collateralization increased stepwise with increasing cystatin C quartiles (P for trend < 0.001). After adjusting for age, gender, risk factors for coronary artery disease, GFR and hsCRP, serum cystatin C ≥ 0.97 mg/L remained independently associated with poor collateralization (OR 2.374, 95% CI 1.660 ~ 3.396, P < 0.001). The diagnostic value of cystatin C levels for detecting poor coronary collateralization persisted regardless of age, gender, presence or absence of diabetes, hypertension or renal dysfunction.

**Conclusions:**

Serum cystatin C reflects angiographic coronary collateralization in patients with stable coronary artery disease, and cystatin C ≥ 0.97 mg/L indicates a great risk of poor coronary collaterals.

## Introduction

Coronary collateral vessels are interarterial connections that potentially offer an alternative source of blood supply to a vascular territory subtended by occluded coronary arteries [1, 2]. Well-formed coronary collaterals are capable of providing a minimum perfusion for jeopardized or hibernating myocardium, preserving left ventricular function, and improving clinical prognosis in patients with coronary artery disease [3–5]. Multiple clinical and biochemical factors and inflammatory cytokines have been suggested to promote or to inhibit the formation of coronary collaterals, and collateral growth is also influenced by the severity of coronary artery disease [6–12]. Cystatin C, an endogenous anti-angiogenic factor, was considered as an emerging biomarker in cardiovascular disease and proved to be an important predictor for adverse outcomes among patients with coronary artery disease [13–15]. However, the diagnostic value of serum cystatin C for evaluating coronary collateralization has been largely unclear. Since early detection of poor coronary collateralization may have clinical relevance as cardiovascular mortality associated with coronary artery disease with or without diabetes or chronic kidney disease is significantly higher partly due to impaired coronary collateralization [1–3], it is pertinent to examine the relationship between serum cystatin C and coronary collateralization in patients with stable coronary artery disease. In this study, we hypothesized that elevated cystatin C level is an indicator of poor coronary collateralization in patients with stable coronary artery disease. We selected a unique cohort of patients with stable angina and chronic total occlusion and assessed the presence and degree of coronary collateralization using the Rentrop scoring system, as a severe coronary stenosis especially complete obstruction is a prerequisite for spontaneous collateral recruitment and this angiographic assessment of coronary collaterals is routinely applied in clinical practice [16, 17]. Serum levels of high-sensitivity C-reactive protein (hsCRP) were also determined to compare inflammatory condition in these patients.

## Subjects and Methods

### Ethics Statement

This study was approved by the Institutional Review Board of Rui Jin Hospital, Shanghai Jiaotong University School of Medicine. Written informed consents were obtained from all patients, and clinical investigation was conducted according to the principle of the Declaration of Helsinki.

### Subjects

**Fig 1:**
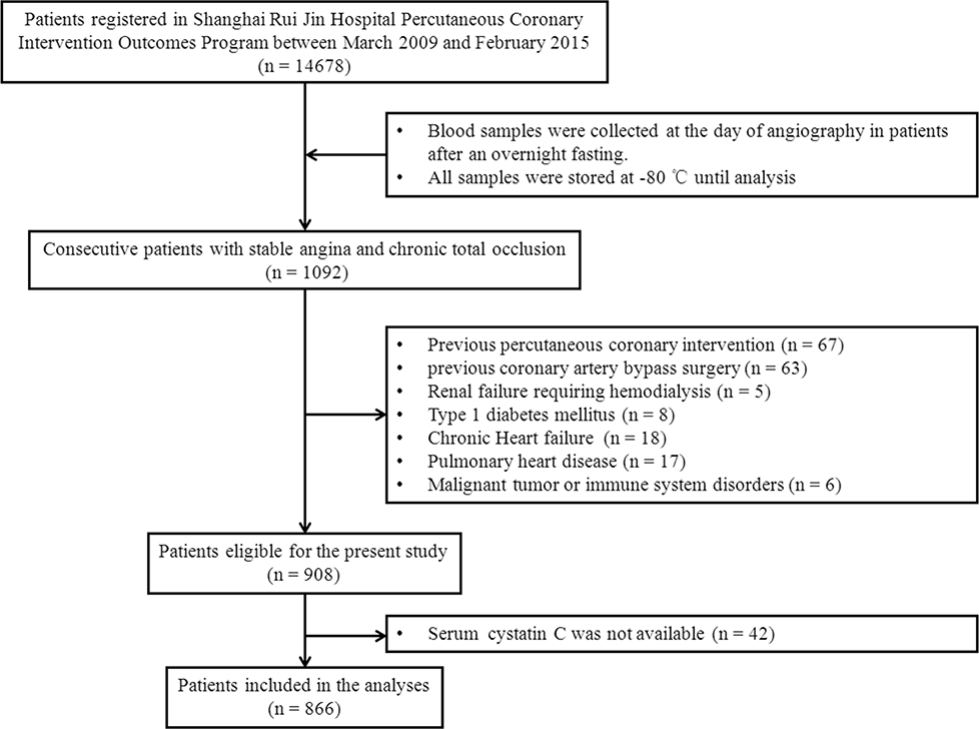
Flowchart of patient enrollment.

Initially, we screened a total of 1092 consecutive patients with stable angina and chronic total occlusion (> 3 months) of at least one major epicardial coronary artery between March 2009 and February 2015 from the database of Advanced Glycation Endproducts and Development of CAD Program (AGENDA, **S1 Document**) in Rui Jin Hospital, Shanghai. This program included coronary artery disease patients with or without diabetes, aiming to investigate the mechanisms of atherosclerosis and elements affecting this process including advanced glycation endproducts in diabetes. Two-hundred and twenty six patients were excluded by the following exclusion criteria: (a) percutaneous coronary intervention within the prior 3 months; (b) previous coronary artery bypass grafting; (c) renal failure requiring hemodialysis; (d) chronic heart failure, pulmonary heart disease, malignant tumor or immune system disorders; (e) type 1 diabetes; and (f) unavailability of cystatin C data. Finally, 866 patients fit the inclusion criteria and composed the study cohort **(Fig 1)**.

Hypertension, type 2 diabetes and dyslipidemia were defined according to European Society of Hypertension (ESH) / European Society of Cardiology (ESC) guidelines for the management of arterial hypertension [18], criteria of the American Diabetes Association [19], and Third Report of The National Cholesterol Education Program (NCEP) [20], respectively. Stable angina was diagnosed based on the criteria of the American College of Cardiology/ American Heart Association [21].

### Angiography and Collateral Grading

Coronary angiography was performed via the femoral or radial access with 6Fr diagnostic catheters. The angiographic assessment was done independently by two blinded interventional cardiologists (RYZ, QZ), according to lesion classification scheme of the American College of Cardiology/ American Heart Association [22]. In case of disagreement, the difference in interpretation was resolved by a third reviewer (WFS). The Rentrop scoring system was used to grade collateral filling from the contra-lateral vessel (often via connections of the epicardial surface or intraventricular septum) as described previously [17]: 0 = none; 1 = filling of side branches only; 2 = partially filling of the epicardial segment; 3 = complete filling of the epicardial segment. Poor coronary collateralization was defined as Rentrop score 0 or 1 and good coronary collateralization was defined as Rentrop score 2 or 3, as in previous studies [6–10]. In patients with more than one chronic total occlusion, the vessel with the highest collateral grade was chosen for analysis.

### Biochemical Measurement and Estimation of GFR

We obtained blood samples at the day of angiography in all patients after an overnight fasting, and stored them at -80°C until analysis. Assessment of serum levels of creatinine, blood urea nitrogen, uric acid, lipid profiles, glucose, and glycosylated hemoglobin (HbA1c), was made with standard laboratory techniques [7, 8]. Serum cystatin C was determined by high sensitive latex-enhanced immune- turbidimetric method with an automatic biochemical analyzer (7600–020; Hitachi Inc, Tokyo, Japan) [23, 24], and hsCRP level was assayed by ELISA (Biocheck Laboratories, Toledo, OH, USA). Glomerular filtration rate (GFR) was estimated using the creatinine-based abbreviated Modification of Diet in Renal Disease (MDRD) formula [25].

### Statistical Analysis

EpiData Entry 3.1 Software was used for data entry and documentation, and EXCEL was adopted to manage database. SPSS 20.0 for Windows (SPSS, Inc., Chicago, IL, USA) was used for statistical analyses. Data are presented as mean ± standard deviation (SD) and number (percentages). For continuous variables, differences between groups were evaluated by t test for normally distributed values; otherwise, the Mann-Whitney U test was applied. For categorical variables, differences between groups were evaluated with the chi-square test. Spearman’s rho test was used to assess the correlation between serum cystatin C and Rentrop scores. The serum levels of cystatin C were divided into 4 groups according to quartile distribution. Univariable and multivariable logistic regression analyses after adjustment for age, gender, body mass index (BMI), traditional risk factors for coronary artery disease including smoking, hypertension, hyperlipidemia and diabetes, multi-vessel disease, GFR, and hsCRP were performed to detect the relationship between poor collateralization and serum levels of cystatin C. Regression models with above-mentioned potential confounding factors (model 1) and further inclusion of serum cystatin C (model 2) were also developed to explore the independent determinants for poor collateralization. Hosmer–Lemeshow X^2^ test was used for model calibration. Receiver-operating characteristic (ROC) analyses were performed with serum cystatin C and predicted probabilities (C statistic) for poor collateralization derived from regression models with and without cystatin C. The areas under the curves (AUC) were compared using DeLong method with MedCalc software for windows (version 11.4, Mariakerke, Belgium). The likelihood ratio test and the net reclassification improvements (NRI) and integrated discrimination improvements (IDI) were used to assess the improvement of goodness of fit and predictive performance for model 2 compared with model 1 [26]. All analyses used 2-sided tests with an overall significance level of alpha = 0.05.

## Results

### Baseline Characteristics

Among overall 866 patients included in the final analysis, poor and good coronary collateralization was detected in 344 and 522 patients, respectively. Patients with poor coronary collateralization were older, females and cigarette smokers in higher percentage and had more diabetes and dyslipidemia but were less hypertensive than those with good collateralization (for all comparisons, P < 0.01). Despite similar degree of coronary artery disease and medical treatments, diastolic blood pressure, high-density lipoprotein cholesterol, and GFR were lower, but serum levels of creatinine and hsCRP were more elevated in those with poor collateralization (**Table 1)**.

**Table 1:** Baseline characteristics and biochemical assessment in patients with poor and good collateralization.

Variables	Poor collateralization (n = 344)	Good collateralization (n = 522)	P value
Female, n (%)	99 (28.8)	84 (16.1)	< 0.001
Age, y	68.2 ± 9.8	62.9 ± 11.0	< 0.001
Body mass index, Kg/m^2^	25.2 ± 3.4	25 ± 3.3	0.301
Smoke, n (%)	150 (43.6)	175 (33.5)	0.003
Diabetes, n (%)	218 (63.4)	280 (53.6)	0.005
Hypertension, n (%)	213 (61.9)	383 (73.4)	< 0.001
Dyslipidemia, n (%)	120 (34.9)	116 (22.2)	< 0.001
Systolic blood pressure, mm Hg	140 ± 20.7	138.8 ± 19.3	0.380
Diastolic blood pressure, mm Hg	82.3 ± 12.1	85.1 ± 12.3	0.001
Severity of CAD, n (%)			0.699
1-vessel	61 (17.7)	93 (17.8)	0.975
2-vessel	99 (28.8)	137 (26.2)	0.413
3-vessel	184 (53.5)	292 (55.9)	0.478
Fasting blood glucose, mmol/L	5.9 ± 2.0	5.6 ± 1.6	0.016
HbA1c, %	6.6 ± 1.4	6.3 ± 1.5	0.022
Triglyceride, mmol/L	1.75 ± 0.88	1.63 ± 0.82	0.047
Total cholesterol, mmol/L	4.33 ± 1.11	4.16 ± 1.09	0.024
HDL cholesterol, mmol/L	0.94 ± 0.25	0.99 ± 0.25	0.002
LDL cholesterol, mmol/L	2.48 ± 0.88	2.48 ± 0.91	0.983
Blood urea nitrogen, mmol/L	5.9 ± 1.9	5.5 ± 1.8	0.001
Uric acid, μmol/L	342 ± 82	334 ± 90	0.190
Creatinine, μmol/L	91.25 ± 23.31	81.32 ± 28.59	< 0.001
Cystatin C, mg/L	1.08 ± 0.32	0.90 ± 0.34	< 0.001
GFR, mL/min/1.73m^2^	79.39 ± 20.45	98.22 ± 27.88	< 0.001
hsCRP, mg/L	5.60 ± 3.78	4.08 ± 3.61	< 0.001
Medication, n (%)			
ACE inhibitor/ARB	161 (46.8)	235 (45.0)	0.606
β-blocker	141 (41.0)	185 (35.4)	0.099
Calcium channel blocker	91 (26.5)	131 (25.1)	0.654
Nitrate	187 (54.4)	284 (54.4)	0.989
Statin	220 (64.0)	347 (66.5)	0.445
Antidiabetic therapy	175 (50.9)	237 (45.4)	0.115

Data are mean ± SD or number (%). ACE, angiotensin converting enzyme; ARB, angiotensin receptor blocker; CAD, coronary artery disease; GFR, glomerular filtration rate; HbA1c, glycosylated hemoglobin A1c; HDL, high-density lipoprotein; hsCRP, high sensitive C reactive protein; LDL, low-density lipoprotein

### Serum Cystatin C and Coronary Collateralization

**Fig 2:**
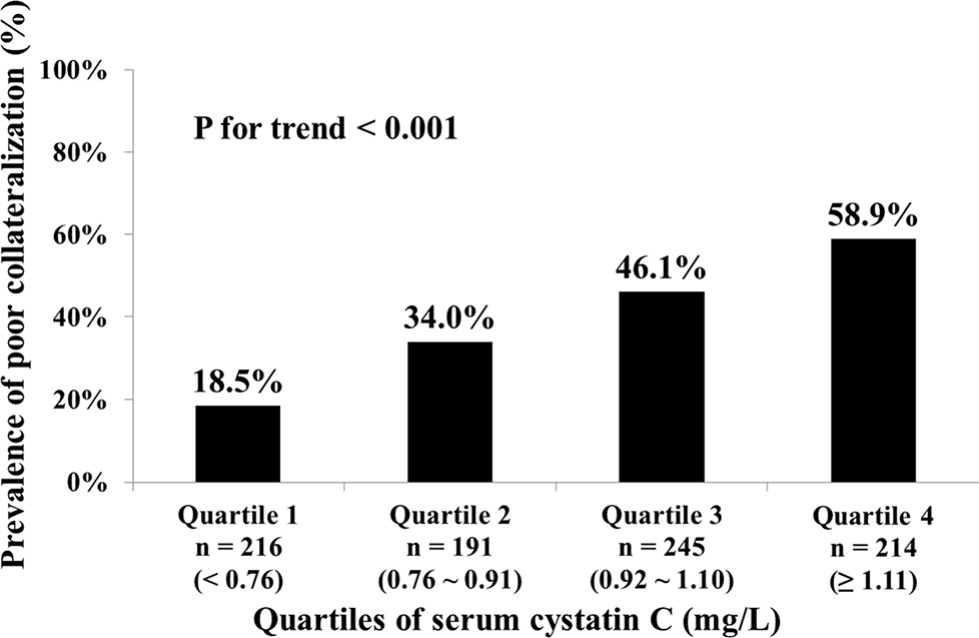
Prevalence of poor collateralization in patients across quartiles of serum cystatin C.

Serum cystatin C was significantly higher in patients with poor collateralization than in those with good collateralization (1.08 ± 0.32 mg/L vs. 0.90 ± 0.34 mg/L, P < 0.001), and correlated inversely with Rentrop score before (Spearmen’s r = -0.263, P < 0.001) and after (Spearmen’s r = -0.145, P < 0.001) adjusting for gender, age, BMI, traditional risk factors for coronary artery disease (including smoking, hypertension, hyperlipidemia and diabetes), multi-vessel disease, GFR and serum level of hsCRP. In addition, the prevalence of poor coronary collaterals increased stepwise from the lowest quartile (< 0.76 mg/L) to the highest quartile of serum cystatin C (≥ 1.11 mg/L) (P for trend < 0.001) **(Fig 2)**. Odds ratio for poor collateralization increased to 6.300 (95% confidence interval [CI] 4.065 ~ 9.764) in the highest compared with those in the lowest quartile of cystatin C level (P < 0.001). These associations remained significant after adjusting for multiple variables (OR: 7.021, 95% CI 4.261 ~ 11.570, P < 0.001) **(Table 2)**.

**Table 2:** Odds ratio of poor collateralization according to cystatin C in patients with chronic total occlusion.

Quartiles of cystatin C (n, range)	Poor collateralization, n (%)	Crude OR (95% CI)	^a^Adjusted OR (95% CI)
Quartile 1 (n = 216, < 0.76 mg/L)	40 (18.5)	1	1
Quartile 2 (n = 191, 0.76 ~ 0.91 mg/L)	65 (34.0)	2.270 (1.439 ~ 3.579) *	1.946 (1.187 ~ 3.190)**
Quartile 3 (n = 245, 0.92 ~ 1.10 mg/L)	113 (46.1)	3.767 (2.462 ~ 5.764) *	3.424 (2.140 ~ 5.477)*
Quartile 4 (n = 214, ≥ 1.11 mg/L)	126 (58.9)	6.300 (4.065 ~ 9.764) *	7.021 (4.261 ~ 11.570)*
Per quartile	/	1.811 (1.583 ~ 2.071)	1.898 (1.622 ~ 2.220)
P value for quartile trend	< 0.001	< 0.001	< 0.001

CI, confidence interval; OR, odds ratio.

*P < 0.001

**P < 0.01 vs quartile 1

^a^Multiple-adjustment for gender, age, body mass index, diabetes, hypertension, dyslipidemia, smoke, multi-vessel disease, glomerular filtration rate and serum level of high sensitive C reactive protein

ROC curve analysis showed that AUC was 0.687 (95% CI 0.652 ~ 0.722, P < 0.001) and the optimal cut-point of serum cystatin C was 0.97 mg/L, with a diagnostic sensitivity and specificity of 61.3% and 67.2% for the presence of poor coronary collateralization.

### Risk for Poor Collateralization

**Fig 3:**
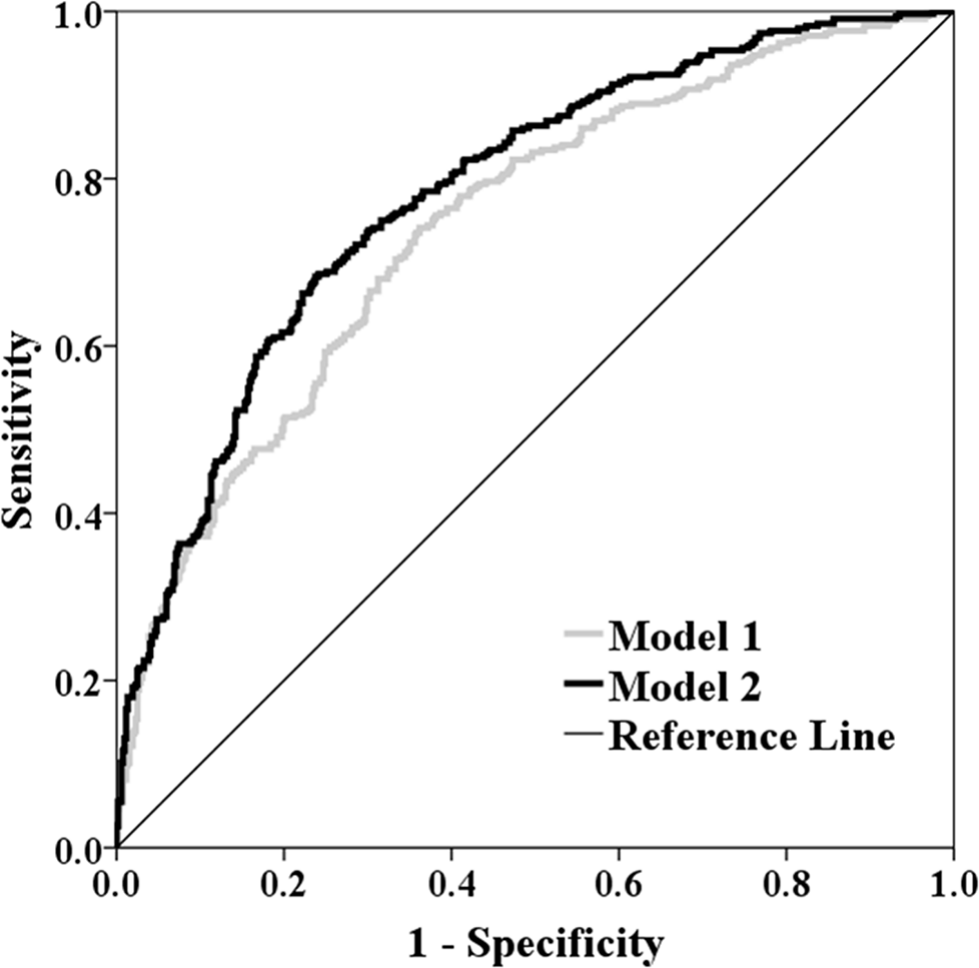
Receiver operating characteristic curves of predicted probabilities derived from regression models for detecting poor collateralization. Model 1 includes variables of age, gender, body mass index, traditional risk factors for coronary artery disease (diabetes, hypertension, dyslipidemia and smoke), multi-vessel disease, GFR and serum level of high sensitive C reactive protein. Model 2 includes variables in model 1 and serum cystatin C ≥ 0.97 mg/L.

Multivariate logistic regression analysis revealed that age, gender, traditional risk factors for coronary artery disease, GFR and serum level of hsCRP were determinants for poor coronary collateralization (model 1). After adjustment for these variables, serum cystatin C ≥ 0.97 mg/L remained independently associated with poor collateralization (OR: 2.374, 95% CI 1.660 ~ 3.396, P < 0.001) (model 2) **(Table 3)**. The calibrations of both models were good (P = 0.480 and P = 0.791, respectively). Compared with model 1, the addition of serum cystatin C (≥ 0.97 mg/L) in model 2 significantly improved the goodness-of-fit and predictive performance with an increase of Nagelkerke R^2^ of 7.3% (P < 0.001) and C statistic of 0.039 (95% CI 0.020 ~ 0.059, P < 0.001) **(Fig 3)** and a NRI and IDI of 10.5% (95% CI 4.6% ~ 16.4%, P < 0.001) and 5.9% (95% CI 4.3% ~ 7.5%, P < 0.001), respectively.

**Table 3:** Logistic regression analyses for poor collateralization in patients with chronic total occlusion.

	Variables	OR (95% CI)	P value
Model 1	Female	0.532 (0.360 ~ 0.787)	0.002
Nagelkerke R^2^ = 0.232	Age	1.597 (1.328 ~ 1.921)	< 0.001
Hosmer-Lemeshow test: P = 0.480	Body mass index	1.172 (0.995 ~ 1.380)	0.057
	Diabetes	1.609 (1.165 ~ 2.222)	0.004
	Hypertension	0.535 (0.380 ~ 0.752)	< 0.001
	Dyslipidemia	1.888 (1.326 ~ 2.687)	< 0.001
	Smoke	2.112 (1.506 ~ 2.961)	< 0.001
	Multi-vessel disease	0.906 (0.593 ~ 1.383)	0.647
	GFR	0.471 (0.388 ~ 0.572)	< 0.001
	hsCRP	1.470 (1.257 ~ 1.719)	< 0.001
Model 2	Female	0.517 (0.347 ~ 0.770)	0.001
Nagelkerke R^2^ = 0.305	Age	1.558 (1.292 ~ 1.878)	< 0.001
Hosmer-Lemeshow test: P = 0.791	Body mass index	1.140 (0.965 ~ 1.347)	0.123
	Diabetes	1.739 (1.251 ~ 2.417)	0.001
	Hypertension	0.518 (0.366 ~ 0.733)	< 0.001
	Dyslipidemia	2.206 (1.528 ~ 3.185)	< 0.001
	Smoke	2.113 (1.500 ~ 2.975)	< 0.001
	Multi-vessel disease	0.886 (0.576 ~ 1.363)	0.583
	GFR	0.568 (0.463 ~ 0.696)	< 0.001
	hsCRP	1.468 (1.252 ~ 1.721)	< 0.001
	Cystatin C ≥ 0.97 mg/L	2.374 (1.660 ~ 3.396)	< 0.001

CI, confidence interval; GFR, glomerular filtration rate; hsCRP, high sensitive C reactive protein; OR, odds ratio

### Diagnostic Value of Cystatin C in Different Patient Subgroups

We performed the sensitivity analyses according to age, sex, and presence or absence of overweight or obesity (BMI ≥ 25 Kg/m^2^), diabetes, hypertension and at least mild renal dysfunction (GFR < 90 mL/min/1.73m^2^) **(Table 4)**. The diagnostic value of cystatin C for detecting poor coronary collateralization was consistent in all patient subgroups, with the AUCs ranging from 0.585 to 0.732 (all P < 0.01). After adjustment for potential confounding factors, serum cystatin C ≥ 0.97 mg/L was independently associated with poor coronary collateralization (OR: 2.630 ~ 5.484, all P < 0.01). The calibrations of all models with and without cystatin C were good (P ≥ 0.125). Compared with the models with traditional variables, additional inclusion of serum cystatin C ≥ 0.97 mg/L also significantly improved the goodness-of-fit and predictive performance in all subgroup analyses with an increase of Nagelkerke R^2^ of 2.9% ~ 10.9% (all P < 0.01), C statistic of 0.029 ~ 0.058 (all P < 0.05), and a NRI and IDI of 8.0% ~ 18.2% (all P < 0.05) and 3.7% ~ 9.6% (all P < 0.001), respectively **(Table 4)**.

**Table 4:** The diagnostic value of serum cystatin C for evaluation of poor collateralization in different patients subgroups.

	n	Poor, n (%)	^a^AUC (95% CI)	^a^Se,%	^a^Sp,%	^b^Adjusted OR (95% CI)	^c^ΔNagelkerke R^2^	^c^ΔC statistic (95% CI)	^c^NRI (95% CI)	^c^IDI (95% CI)
Overall	866	344 (39.7)	0.687 (0.652 ~ 0.722)*	61.3	67.2	3.507 (2.521 ~ 4.878)*	0.073#	0.039 (0.020 ~ 0.059)#	10.5% (4.6% ~ 16.4%)#	5.9% (4.3% ~ 7.5%) #
Female	183	99 (54.1)	0.732 (0.660 ~ 0.805) *	65.7	73.8	5.484 (2.542 ~ 11.830)*	0.109#	0.054 (0.012 ~ 0.097)###	18.2% (4.6% ~ 31.9%)##	9.6% (5.3% ~ 13.8%)#
Male	683	245 (35.9)	0.676 (0.636 ~ 0.717) *	59.6	66.0	3.120 (2.155 ~ 4.517)*	0.064#	0.039 (0.016 ~ 0.062)##	7.4% (0.5% ~ 14.4%)###	5.0% (3.3% ~ 6.7%)#
Age < 65 y	418	118 (28.2)	0.696 (0.643 ~ 0.749) *	52.5	73.3	3.736 (2.281 ~ 6.120)*	0.088#	0.051 (0.008 ~ 0.093)###	12.2% (1.8% ~ 22.6%)###	6.4% (3.6% ~ 9.1%)#
Age ≥ 65 y	448	226 (50.4)	0.645 (0.594 ~ 0.697) *	65.9	59	3.891 (2.478 ~ 6.110)*	0.091#	0.052 (0.021 ~ 0.082)#	13.2% (4.5% ~ 21.8%)##	7.4% (5.0 ~ 9.8%)#
BMI < 25 Kg/m^2^	447	168 (37.6)	0.673 (0.623 ~ 0.723) *	62.5	66.7	3.222 (2.030 ~ 5.114)*	0.063#	0.035 (0.009 ~ 0.062)##	8.6% (0.3% ~ 16.9%)###	6.9% (4.2% ~ 9.6%)#
BMI ≥ 25 Kg/m^2^	419	176 (42.0)	0.703 (0.654 ~ 0.752) *	60.2	67.9	4.002 (2.465 ~ 6.497)*	0.087#	0.042 (0.011 ~ 0.072)##	12.6% (4.3% ~ 21.0%)##	5.6% (3.5% ~ 7.6%)#
Non-diabetes	368	126 (34.2)	0.685 (0.628 ~ 0.741) *	68.3	61.6	4.368 (2.513 ~ 7.591)*	0.090#	0.044 (0.012 ~ 0.078)##	8.8% (0.9% ~ 16.7%)###	6.9% (4.2% ~ 9.6%)#
Diabetes	498	218 (43.8)	0.706 (0.661 ~ 0.751) *	60.9	73.2	3.202 (2.099 ~ 4.886)*	0.067#	0.041 (0.015 ~ 0.067)##	11.7% (3.5% ~ 20.0%)##	5.6% (3.5% ~ 7.6%)#
Non-hypertension	270	131 (48.5)	0.689 (0.627 ~ 0.752) *	57.3	70.5	3.112 (1.716 ~ 5,646)*	0.057#	0.036 (0.006 ~ 0.067)###	14.1% (3.5% ~ 24.7%)##	5.4% (2.9% ~ 7.9%)#
Hypertension	596	213 (35.7)	0.691 (0.648 ~ 0.734) *	63.8	66.1	3.678 (2.463 ~ 5.492)*	0.081#	0.058 (0.031 ~ 0.085)#	8.0% (0.6% ~ 15.4%)###	7.7% (5.6% ~ 9.7%)#
GFR ≥ 90 mL/min/1.73m^2^	408	97 (23.8)	0.667 (0.609 ~ 0.725) *	32.0	83.3	2.630 (1.422 ~ 4.863)**	0.029##	0.029 (0.007 ~0.050)##	12.8% (1.4% ~ 24.2%)###	3.7% (1.8% ~ 5.7%)#
GFR < 90 mL/min/1.73m^2^	458	247 (53.9)	0.585 (0.532 ~ 0.639)**	72.9	43.6	2.787 (1.772 ~ 4.382)*	0.050##	0.043 (0.018 ~ 0.068)##	12.0% (4.1% ~ 20.0%)##	5.0% (3.2% ~ 6.8%)#

AUC, area under curve; BMI, body mass index; CI, confidence interval; IDI, integrated discrimination improvement; GFR, glomerular filtration rate; NRI, net reclassification improvement; OR, odds ratio

a AUC,Se and Sp denote area under the curve for serum cystatin C in detecting poor collateralization in different patients subgroup and corresponding sensitivity and specificity with a cut-off point of 0.97 mg/L

b odds ratio (95% CI) of serum cystatin C ≥ 0.97 mg/L after adjustment for gender, age, BMI, diabetes, hypertension, dyslipidemia, multi-vessel disease and serum level of high sensitive C reactive protein (hsCRP).

c Improvement of goodness-of-fit and predictive performance of additional inclusion of serum cystatin C ≥ 0.97 mg/L to the model with traditional variables including gender, age, BMI, diabetes, hypertension, dyslipidemia, multi-vessel disease and hsCRP

*P < 0.001

**P < 0.01 for AUC or adjusted OR of cystatin C in diagnosing poor collateralization

#P < 0.001

##P < 0.01

###P < 0.05 for comparisons between logistic regression models with and without serum cystatin C ≥ 0.97 mg/L

## Discussion

This study showed that elevated serum cystatin C was associated with poor coronary collateralization in patients with stable coronary artery disease and chronic total occlusion.

It is well recognized that after coronary artery occlusion, myocardial blood flow distal to occluded segment could be, at least partly, restored by collateral formation mainly through two different processes of blood vessel growth. New capillaries may form via ischemia-induced sprouting from post-capillary venules (angiogenesis), and the development and maturation of these newly growing vessels are regulated by a balance of pro-angiogenic and anti-angiogenic factors. Arteriogenesis denotes a transformation of pre-existing arterioles into functional (muscular) collateral arteries and is predominantly stimulated by pressure gradient across the occluded segment and tangential fluid shear stress at the vessel wall. Arteriogenesis, not angiogenesis, is the leading mechanism in restoring blood flow following arterial occlusion. Both processes are related to endothelial and smooth muscle cells and various growth factors and adversely affected by inflammatory cytokines [27]. Collateral growth in patients with coronary artery disease is highly heterogeneous and influenced by a number of factors. We found that old age, female gender, traditional risk factors for coronary artery disease, and reduced renal function were associated with poor collateralization, whereas hypertension, especially elevated diastolic blood pressure, correlated with well-formed coronary collaterals. Several studies have suggested that poorly developed coronary collaterals may be related to chronic inflammation of low degree, as evidenced by elevated serum levels of cytokines such as hsCRP [28], tumor necrosis factor (TNF)-α [29], soluble endothelial adhesion molecules [30], monocyte chemoattractant protein-1 [31], and C1q/TNF-related protein-1 [8], which inhibit key components of angiogenesis particularly endothelial progenitor cell differentiation, survival and function. Recently, apelin has been shown to promote angiogenesis, and its serum levels were associated with coronary collateral development [32]. Akin et al revealed that white blood cell subtypes, especially the neutrophil-to-lymphocyte ratio, can be used as an indicator of systemic inflammation, and an elevated neutrophil- to-lymphocyte ratio was independently associated with impaired coronary collateral formation in patients with stable coronary artery disease [9]. High serum cystatin C was thought to be related directly to both inflammation and atherosclerosis [13]. Some studies have highlighted that inflammatory cytokines stimulate lysosomal cathepsins, which may be associated with increased cystatin C levels [33]. These effects could cause worse vascular endothelial changes, inflammation and atherosclerosis in patients with coronary artery disease. However, the role of cystatin C in the formation of coronary collaterals remains largely unclear. The present study demonstrated that poor coronary collateralization corresponded significantly with elevated cystatin C levels, which is contrast to a previous report showing no significant difference in serum cystatin C between patients with poor and good coronary collaterals [34]. The exact explanation behind these different results may be, at least partly, related to a heterogeneous population in their study, which contained a small number of diabetic patients (< 20% in the final analysis) and a significant proportion of patients with acute coronary syndrome (approximately 50%) or non-occluded coronary lesions [34]. It is well documented that hyperglycemia abolished the formation of coronary collateral vessels [35], and diabetes represents an independent risk factor for poor coronary collateralization [10, 36]. In addition, cysteine proteases are associated with formation of necrotic core and rupture of the cap, as well as macrophage apoptosis in atherosclerotic plaques, and the synthesis of cystatin C may be significantly increased during acute myocardial ischemia [37]. Notably, the recruitment of coronary collaterals is predominantly driven by shear force along the pressure gradient that develops when the native vessel is tightly stenotic or even completely occluded [16, 38]. In view of these conditions and for avoiding possible confounding factors, we analyzed the relationship between serum cystatin C and coronary collateralization in a unique cohort of patients with stable angina and chronic total occlusion.

The main finding of this study is that serum cystatin C correlated inversely with Rentrop score even after adjusting for multiple variables including GFR and levels of hsCRP. The prevalence of poor coronary collateralization increased stepwise with increasing cystatin C quartiles. An optimal cut-off point of serum cystatin C level of 0.97 mg/L provided a diagnostic sensitivity and specificity of 61.3% and 67.2% for the presence of poor coronary collateralization. Patients with a cystatin C ≥ 0.97mg/L had 2.37-fold increased risk of poor collateralization. Interestingly, additional inclusion of serum cystatin C ≥ 0.97 mg/L in the model significantly improved the diagnostic accuracy for poor coronary collateralization in all subgroup population based on age, gender, presence or absence of diabetes, hypertension, or at least mild renal dysfunction by 8.0–18.2%. These observations support a notion that anti-angiogenic cystatin C is an indicator of coronary collateral formation in patients with stable coronary artery disease and chronic total occlusion. The results of the present study are also in line with our previous findings that cystatin C-based equation was superior to creatinine-based formula for estimating GFR and identifying poor collateralization in type 2 diabetic patients with coronary artery disease [24]. Because available data on cystatin C-based GFR equation remain very limited, and measurement of cystatin C is inexpensive and easily available, the possible use of serum cystatin C as a marker for risk of poor coronary collateralization would be desirable and clinically meaningful in the management of stable coronary artery disease patients.

It is evident that the effect of cystatin C levels on coronary collateralization reflects the net result of several pathophysiological processes. Cystatin C *per se* displays anti-angiogenic characteristics by reducing endothelial cell tubule formation and cysteinyl cathepsin activities [37, 39]. Furthermore, cystatin C as a cysteine proteinase inhibitor is associated with cardiovascular risk factors as well as inflammation, which may promote vascular endothelial damage and cause poor collateralization [13, 15]. Notably, approximately 60% of patients had type 2 diabetes in the present study. Previous reports including ours have shown that an interaction between advanced glycation endproducts (AGE) and receptor for AGE (RAGE) plays a critical role in the acceleration of coronary atherosclerosis and abnormal collateral vessel formation [10,35,36]. Likewise, renal dysfunction which commonly occurs in patients with severe coronary artery disease and is partly reflected by elevated serum levels of cystatin C, adversely affect several components necessary for collateral growth through various regulatory mechanisms and gene expression [40–44].

The development of coronary collaterals may be also influenced by medications. It has been shown that statin treatment was associated with good collateralization assessed by the Rentrop classification due partly to reduced apoptosis [45], whereas angiotensin-converting enzyme inhibitor (ACEI) therapy contributed to poor collateral development in patients with coronary occlusion via inhibiting the expression of angiotensin II-induced growth factors such as vascular endothelial growth factor, platelet-derived growth factor, and fibroblast growth factor [46]. Recently, van der Hoeven et al reported a positive relation between chronic use of βblockers and well-formed collaterals assessed by collateral flow index [47]. The use of βblockers reduces heart rate and improves fluid shear stress at the endothelial wall which stimulates coronary collateral growth. In addition, βblockers could decrease catecholamine-mediated inflammatory response. In this study we did not observe an association between medical treatments with statins, ACEI or βblockers and angiographic grade of coronary collateralization.

Although the Rentrop scoring system is the most widely used angiographic mean for grading coronary collateralization in routine clinical practice, it shows only a weak correlation with invasive parameters of collateral function and does not actually rate the collaterals themselves, but their effect in filling the occluded arterial segment [17]. Furthermore, adequate angiographic plane and optimal axial alignment of the catheter during contrast injection and acquisition time of collateral filling are important for assessing collateral circulation using the Rentrop method. Measurement of coronary flow index which requires simultaneous recordings of central aortic pressure and the distal pressure within the occluded segment of the coronary artery represents the “gold standard” for evaluating the competence and functional significance of coronary collaterals. However, coronary flow index can only be determined during coronary intervention, and meticulous care should be taken to keep the guiding catheter away from the coronary orifice during aortic pressure recording and to ensure the absence of antegrade flow leakage after pressure wire passage.

One of the limitations is that the study design was one of retrospective analysis for the point of coronary collateral investigation; thus, it allows us to detect association but not to predict outcome. Nevertheless, these results may reflect true associations in the real world setting as prescription data were captured from a standard database. Another limitation is that our study did not include other possible factors influencing collateral development in patients with coronary artery disease especially inflammatory cytokines [8–12, 28–31], although the effects of the established clinical variables were evaluated by multivariate analysis. The relative strength of a model by incorporating all potential biomarkers for predicting poor coronary collateralization deserves further investigations.

In conclusion, the present study is the first to demonstrate an association between elevated serum cystatin C and reduced angiographic coronary collateralization in a large cohort of stable coronary disease patients with chronic total occlusion. The optimal cut-point of cystatin C in serum of ≥ 0.97 mg/L indicates a great increased risk of poor coronary collateralization. These findings may provide new insights on risk assessment and management strategy for this unique high-risk population.

## Supporting Information

S1 Document Protocol Registration Receipt: Advanced Glycation Endproducts and Development of CAD (AGENDA) (NCT02089360). (DOCX)
