# Supplementary material for: Serum Cystatin C Reflects Angiographic Coronary Collateralization in Stable Coronary Artery Disease Patients with Chronic Total Occlusion
Source: PLoS One. 2015 Sep 24;10(9):e0137253. doi: 10.1371/journal.pone.0137253 (PMC4581619; doi:10.1371/journal.pone.0137253)
Supplement: S1 Document — (DOCX) [file pone.0137253.s001.docx]

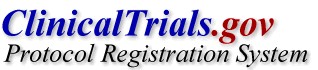

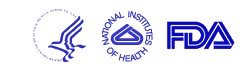


## Protocol Registration Receipt

03/14/2014

Adavanced Glycated Endproducts and Development of CAD (AGENDA)

This study is currently recruiting participants.

Verified by Zhang Qi, MD, Shanghai Jiao Tong University School of Medicine, March 2014

| Sponsor: | Shanghai Jiao Tong University School of Medicine |
| --- | --- |
| Collaborators: |  |
| Information provided by (Responsible Party): | Zhang Qi, MD, Shanghai Jiao Tong University School of Medicine |
| ClinicalTrials.gov Identifier: | NCT02089360 |


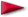
 Purpose

Blood sample is prospectively taken from consecutive patients underwent coronary angiogram in our center, after getting informed consent from the patients. Serum level of advanced glycation end products (AGEs) was measured and the clinical features of patients (including angiographic results) were entered into our database.

Clinical follow-up was performed for all patients, and the relationship between AGEs and paitents’ outcome were analyzed. Further intervention will be adjusted according to the results,including clinical and basic research in lab.

Condition

Atherosclerosis Inflammation

Study Type: Observational [Patient Registry] Study Design: Cohort, Prospective

Target Follow-Up Duration: 5 Years

Official Title: Study of Serum Level of Advanced Glycated Endproducts and Development of Coronary Atherosclerosis in Patients Undergoing Coronary Angiogram

## Further study details as provided by Zhang Qi, MD, Shanghai Jiao Tong University School of

Medicine:

Biospecimen Retention: Samples With DNA

Blood sample is taken for all patients underwent coronary angiogram after getting informed consent, and stored in requested condition, for measuring serum levels of different kinds of factors.

Primary Outcome Measure:

- MACE [Time Frame: 5-year] [Designated as safety issue: No]

including death, occurrence of myocardial infarction, and coronary revascularization

Secondary Outcome Measures:

- progression in coronary atherosclerosis [Time Frame: 5-year] [Designated as safety issue: No] repeated coronary angiogram in patients with symptom aggravation or occurrence of myocardial infarction, to evaluate the progression of coronary lesions, comparing to the baseline results.

Other Pre-specified Outcome Measures:

- Serum level of AGEs [Time Frame: baseline] [Designated as safety issue: No]

serum levels of AGEs will be measured and the relationship between clinical outcomes will be analyzed.

Estimated Enrollment: 10000 Study Start Date: January 2014

Estimated Primary Completion Date: January 2016

Number of arms: 1

Serum levels of HMGB1, HMGB2, ADAM10, etc. will be measured in lab and animal studies will be designed to intervene the adverse interaction between abnormal serum concentration of AGEs, etc. and worse clinical results.

#
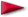
 Eligibility

patients underwent coronary angiogram Sampling Method: Probability Sample

Ages Eligible for Study: 18 Years to 90 Years

Genders Eligible for Study: Both

Inclusion Criteria:

- patients underwent coronary angiogram for suspected coronary artery disease and potential percutaneous coronary intervention

Exclusion Criteria:

- patients underwent coronary angiogram for other reasons, including pre-surgery examination
- patients with severe co-morbidity, and life expectancy less than one-year

#
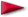
 Contacts and Locations

## Contacts

| Qi Zhang, PhD | 86-21-64370045 Ext. 665380 | [rjheart@gmail.com](mailto:rjheart@gmail.com) |
| --- | --- | --- |
| Wei Feng Shen, PhD | 86-21-64370045 Ext. 665215 | [rjshenweifeng@gmail.com](mailto:rjshenweifeng@gmail.com) |

Locations

China, Shanghai

Ruijin Hospital, Shanghai Jiaotong University School of Medicine Recruiting

Shanghai, Shanghai, China, 200025

Contact: Lin Lu, PhD 86-21-64370045 Ext. 610910 [rjlulin1965@163.com](mailto:rjlulin1965@163.com) Principal Investigator: Lin Lu, PhD

#
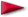
 More Information

Responsible Party: Zhang Qi, MD, Director, Cardiac Catheterization Lab, Shanghai Jiao Tong University School of Medicine

Study ID Numbers: RJH20140311

Health Authority: China: Ethics Committee
